# Death receptor 5 expression is inversely correlated with prostate cancer progression

**DOI:** 10.3892/mmr.2014.2504

**Published:** 2014-08-21

**Authors:** ANGELES HERNANDEZ-CUETO, DANIEL HERNANDEZ-CUETO, GABRIELA ANTONIO-ANDRES, MARISELA MENDOZA-MARIN, CARLOS JIMENEZ-GUTIERREZ, ANA LILIA SANDOVAL-MEJIA, ROSARIO MORA-CAMPOS, CESAR GONZALEZ-BONILLA, MARIO I. VEGA, BENJAMIN BONAVIDA, SARA HUERTA-YEPEZ

**Affiliations:** 1 Immunology and Infection Research Unit, National Medical Center ‘La Raza’, IMSS, Mexico City 02200, Mexico; 2 School of Medicine, UNAM, Mexico City 04530, Mexico; 3 Oncology Disease Research Unit, Children Hospital of Mexico ‘Federico Gomez’, Mexico City 06720, Mexico; 4 Department of Pathology, Hospital General Regional No. 25, IMSS, Mexico City 06720, Mexico; 5 Women Hospital, Mexico City 11340, Mexico; 6 Gineco-Obstetricia Hospital No. 3, CMN ‘La Raza’, IMSS, Mexico City 02200, Mexico; 7 Speciality Hospital CMN ‘La Raza’, IMSS, Mexico City 02200, Mexico; 8 Oncology Research Unit, Oncology Hospital, Siglo XXI National Medical Center, IMSS, Mexico City 06720, Mexico; 9 Department of Microbiology, Immunology and Molecular Genetics, David Geffen School of Medicine, University of California, Los Angeles, CA 90095, USA

**Keywords:** death receptor 5, yin yang 1, prostate cancer, tissue microarray

## Abstract

Prostate carcinoma (PCa) is one of the most common cancers in men. Prostate-specific antigen (PSA) has been widely used to predict the outcome of PCa and screening with PSA has resulted in a decline in mortality. However, PSA is not an optimal prognostic tool as its sensitivity may be too low to reduce morbidity and mortality. Consequently, there is a demand for additional robust biomarkers for prostate cancer. Death receptor 5 (DR5) has been implicated in the prognosis of several cancers and it has been previously shown that it is negatively regulated by Yin Yang 1 (YY1) in prostate cancer cell lines. The present study investigated the clinical significance of DR5 expression in a prostate cancer patient cohort and its correlation with YY1 expression. Immunohistochemical analysis of protein expression distribution was performed using tissue microarray constructs from 54 primary PCa and 39 prostatic intraepithelial neoplasia (PIN) specimens. DR5 expression was dramatically reduced as a function of higher tumor grade. By contrast, YY1 expression was elevated in PCa tumors as compared with that in PIN, and was increased with higher tumor grade. DR5 had an inverse correlation with YY1 expression. Bioinformatic analyses corroborated these data. The present findings suggested that DR5 and YY1 expression levels may serve as progression biomarkers for prostate cancer.

## Introduction

Prostate cancer (PCa) is the second leading cause of cancer-associated mortality in the United States and the first in Mexico among men. PCa is the most commonly diagnosed cancer in males (1,2). Most PCa-associated mortalities are due to advanced disease, and androgen deprivation therapy is considered the primary approach in the treatment of symptomatic advanced prostate cancer (3). This treatment option is palliative rather than curative and although it can marginally improve the likelihood of survival, the majority of all patients progress to hormone-refractory PCa. The rate of PCa is highest among Caucasians and African-Americans. The pathogenesis of PCa is very long and results in frequent hospital visits, which causes a high cost of treatment (4–6).

Although the survival rates for PCa patients are high, because of the slow and steady nature of the disease, 30–40% of patients experience prostate-specific antigen (PSA) recurrence within 10 years of surgery or radiation treatment (7). Furthermore, tumor cells ultimately develop resistance to therapy; however, there are few alternative treatments available. Consequently, the median survival of advanced and recurring disease in patients with prostate cancer is drastically reduced (8).

An increase in circulating PSA levels following definitive therapy usually accurately predicts progressive disease. PSA can have a prognostic significance prior to therapy, since lower PSA levels indicate that the disease will not likely recur and higher levels indicate future resistance to therapy (9). PSA, however, is a problematic screening tool since the overall sensitivity may be too low to effectively predict mortality/survival rates (10). Conversely, increasing the sensitivity of PSA may result in false-positive diagnosis of the disease, since the majority of males with PCa will die of unrelated causes (11). Novel PCa biomarkers are required, which should have the ability to distinguish between a benign and a malignant disease (12,13).

Apoptosis through the activation of two predominant pathways may be induced by the binding of ligands to specific receptors on the cell surface, or by non-specific cellular stress (14). Both pathways converge at the level of the caspases, which mediate cell death via cleavage of various cellular substrates. Death receptors (DR) 4 and 5 bind to the tumor necrosis factor-related apoptosis-inducing ligand (TRAIL), which results in the recruitment of the Fas-associated death domain (FADD) to the intracellular death domain and subsequently induces the cleavage of procaspase-8 which initiates a cascade of events leading downstream to apoptosis (15–18).

Reported studies have demonstrated that the surface expression of DR5 is a potentially useful prognostic marker in various cancers (19). The progression of melanoma has been previously associated with a decrease in DR5 and DR4 expression (20). Furthermore, DR5, along with other TRAIL receptors, was shown to be useful in determining the risk of breast cancer metastasis and patient survival (21). However, other studies have shown that DR5 was not a significant prognostic marker (22,23). These observations suggested that the prognostic value of DR5 may be cancer type-specific. In these cases, the role of DR5 repression in PCa was not examined.

A previous study by our group reported that the transcription factor Yin Yang 1 (YY1) may function in the pathogenesis of PCa. High expression levels of YY1 were associated with tumor progression, and patient survival was linked to lower levels of YY1 (24). In addition, it was previously demonstrated that YY1 may transcriptionally repress DR5 expression in prostate cancer cell lines (25–27).

The present study investigated the expression levels of DR5 in PCa and its clinical significance. Since it was reported that YY1 negatively regulates DR5 expression and that YY1 is overexpressed in PCa (24,25), it was hypothesized that DR5 expression may be inhibited in PCa and may be inversely correlated with YY1 expression. This hypothesis was tested by immunohistochemistry (IHC) on tissue microarray (TMA) constructs, prepared with tumor tissues derived from patients with PCa and PIN.

## Materials and methods

### Patients

The study was approved by the Ethics Committee of the Children Hospital of Mexico ‘Federico Gomez’ (Mexico City, Mexico; HIM/2007/061). The study cohort consisted of samples from 93 randomly selected hormone-naïve patients, who underwent radical retropubic prostasectomy or transurethral resection between 2002 and 2007 from the Department of Pathology of Hospital General Regional No. 25, IMSS and Speciality Hospital CMN ‘La Raza’, IMSS (Mexico City, Mexico). The cohort consisted of 20 low grade PIN low grade (LG PIN), 19 high grade PIN (HG PIN) and 54 PCa samples. The 54 cases of PCa were classified according to the Gleason score as follows: 27 low-grade prostatic carcinoma [Gleason score 2–5 (LG PCa)] and 27 high-grade prostatic carcinoma [Gleason score 6–10 (HG PCa)].

### Prostate TMA construction

Formalin-fixed, paraffin-embedded archival tumor specimens were prepared at the Immunology and Infection Research Unit, National Medical Center ‘La Raza’ and at the Oncology Disease Research Unit, Children Hospital of Mexico ‘Federico Gomez’, SSa. At least three core tissue biopsies (each 0.6 mm in diameter) were taken from morphologically representative regions of each prostate tumor and precisely arrayed as previously described (29–31). Tumor samples were accompanied by matching benign (morphologically normal or hyperplasic) and PIN lesions, where available. Tissues were arrayed into five TMA blocks. For staining, sections (4 μm) were transferred to glass slides using an adhesive slide system (PSA-CS 4; Instrumedics, Inc., St. Louis, MO, USA) to support cohesion of the array elements.

### Immunohistochemical analysis

Slices cut at 4 μm for the TMA were placed on slides and either stained with Mayer’s hematoxylin and eosin (H&E) for histopathological examination, or used for subsequent immunohistochemical analysis. The expression levels of DR5 and YY1 were determined using DR5 (Abcam, Cambridge, MA, USA) and YY1 (Santa Cruz Biotechnology, Inc., Santa Cruz, CA, USA) antibodies. Antigen retrieval was performed by immersing the slides in a solution of 0.01% sodium citrate pH 6.0 for 5 min in boiling water. Endogenous peroxidase activity was inhibited by immersing the slides in 3% H_2_O_2_-methanol and background-unspecific binding was decreased by incubating the slides in 2% bovine serum albumin (BSA; Sigma-Aldrich, St. Louis, MO, USA) in phosphate-buffered saline (PBS) for 60 min. The slides were incubated overnight at room temperature with predetermined optimal concentrations of anti-DR5 polyclonal antibody (1:500) and anti-YY1 polyclonal antibody (1:750). In order to decrease variability, all samples were processed at the same time in a single experiment, using a single batch of antibody diluted in PBS-BSA. Following washing, the slides were incubated with a biotinylated secondary antibody (Universal LSAB kit; Dako Corporation, Carpinteria, CA, USA) for 30 min at room temperature, followed by incubation with a streptavidin-horseradish peroxidase conjugate (Universal LSAB kit) for 30 min at room temperature and then with 3,3′-diaminobenzidine tetra-hydrochloride (liquid DAB, Dako Corporation). The reaction was stopped ny adding distilled water and the slides were counterstained with H&E. The tissue was washed in tap water for 5 min, dehydrated using an ethanol series (70, 90 and 100%) in xylene and mounted with E-2 mounting medium (Shandon lab, Pittsburgh, PA, USA). The slides were then analyzed by light microscopy (Olympus BX-40; Olympus Corporation, Tokyo, Japan).

### Scoring of immunohistochemical staining

A semi-quantitative assessment of tissue antibody staining was conducted by an expert pathologist on prostate analysis, who was blinded to the pathological variables. The stained slides were verified by a second expert to ensure consistency in the scoring. Positive expression was scored based on either a positive staining on the membrane (DR5) or positive staining only in the nucleus (YY1). Data are presented as positively stained target cells per 100 cells (range 0–100% positive), per tissue region in the TMA (4 regions in each slide). In addition, the integrated optical density (IOD) in ~300 μm^2^ regions per sample, selected randomly using the Image Pro-plus 6.4 software (Media Cybernetics, Bethesda, MD, USA), were analyzed.

The analysis of the expression levels on the TMAs was performed in a blind manner. The TMA spot was a second blinded quantitative assessment by the same pathologist. The target tissue for scoring was the glandular prostatic epithelium and the scoring of benign tissues did not include basal cells. The tissue spot histology and grading were confirmed on the counterstained study slides. Positive expression, indicated by a brown color, was quantified using the IODs. The density of the staining intensity in each region was analyzed using the Image Pro-plus 6.2 software (MediaCybernetics, Rockville, MD, USA) that was obtained with the diffusion of the light wavelength through the color density in the cells stained brown. The target tissue for scoring was performed in the malignant cells by considering the nuclear staining pattern for YY1 and the membrane cytoplasmic staining pattern for DR5. YY1 nuclear expression and DR5 cytoplasmic membrane expression were scored using two measures, negative and positive (weakly, moderately and strongly positive) intensities, in target cells. The data are presented as positively stained target cells per 100 cells (range 0–100% positive), per region on each spot (four regions in each spot), or as density, whereby four 100 μm^2^ regions per spot were selected randomly to represent expression within each case. The mean pooled integrated intensity of the tumor or control spots was used.

### Statistical analysis

The data were analyzed using Student’s t-test for parametric data and the Mann-Whitney test for non-parametric data. To analyze normalized data, both the analysis of variance (ANOVA) and the Bonferroni tests were used. P<0.05 was considered to indicate a statistically significant difference.

The optimal cut-point for dichotomized PCa malignancy data was determined using the Gleason scoring system (32). The YY1-IOD-expression and DR5 expression were quantified by determining the sum of the IODs and the mean was calculated for each pathological group (malignant cells). For YY1 nuclear and DR-5 cytoplasmic membrane staining, the percentage of positive malignant cells (weak to strong brown staining) was obtained. Descriptive statistics were gathered for all the assembled data. For differences between the pathological groups that were used, Student’s t test and the ANOVA Pearson’s tests were used to analyze correlations of parametric data, respectively. Box plots to compare the various groups (central tendency, dispersion and symmetry of the data) were generated. P<0.05 was considered to indicate a statistically significant difference. All statistical analyses were performed using SSPS 11 statistical analysis program for Windows (SPSS Inc., Chicago, IL, USA) and GraphPad Prism 5.0 (GraphPad, San Diego, CA, USA).

### Comparative metaprofiling of cDNA expression data

The Oncomine Premium database (Oncomine™ Compendia Bioscience, Ann Arbor, MI, USA) was used for analysis and visualization of the bioinformatics analyses (www.oncomine.com). The differential expression analysis of YY1 and DR5 in existing prostate cancer microarray datasets was analyzed by setting a threshold value for gene rank at 10% and P<0.05.

## Results

### Evaluation of YY1 and DR5 protein expression in human prostate cancer tissues

**Figure 1:**
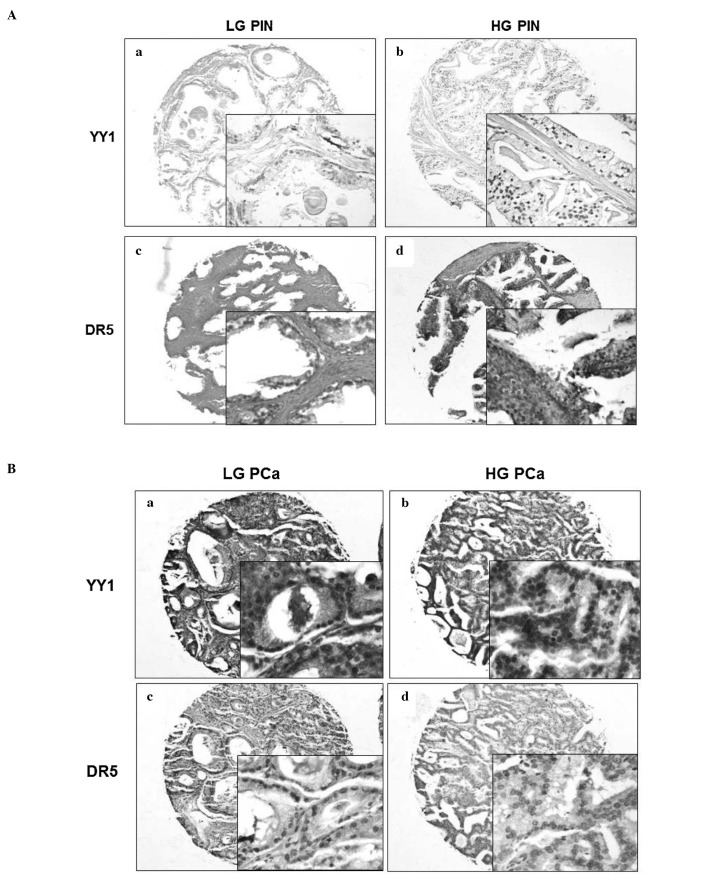
YY1 and DR5 expression in PIN and PCa in tissue microarray constructions. (A) Immunohistochemical staining for YY1 and DR5 in LG PIN and HG PIN samples. (a) LG PIN tissue showed weak nuclear and cytoplasmic epithelial staining of glandular cells. (b) HG PIN tissue showed frequently higher staining. Scoring was derived from the nuclear expression. (c) LG PIN tissue showed high cytoplasmic membrane staining of glandular cells. (d) HG PIN tissue showed frequently high staining. Scoring was derived from the cytoplasmic membrane staining expression. (B) Immunohistochemical staining for YY1 and DR5 in LG PCa and HG PCa samples. (a) LG PCa tissue showed moderate nuclear epithelial staining of glandular cells. (b) HG PCa tissue showed frequently strong nuclear epithelial staining of glandular cells. (c) LG PCa tissue showed weak cytoplasmic membrane staining of glandular cells. (d) HG PCa tissue showed the weakest to absent staining. Scoring was derived from the cytoplasmic staining expression. Magnification, ×100 with ×400 inserts. YY1, Yin Yang 1; DR5, death receptor 5; LG PCa, low grade prostate carcinoma; HG PCa, high grade prostate carcinoma; LG PIN, low grade prostatic intraepithelial neoplasia; HG PIN, high grade prostatic intraepithelial neoplasia.

**Figure 2:**
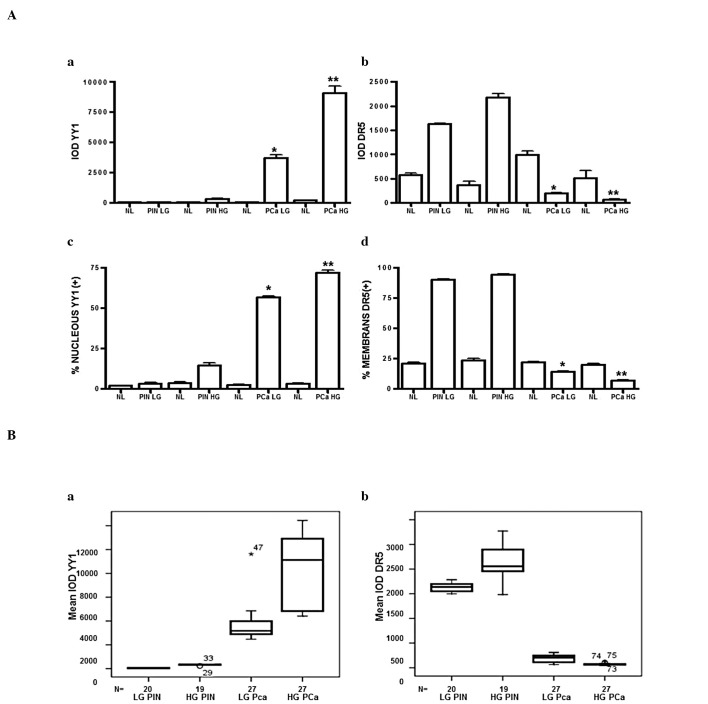
Expression and distribution of YY1 and DR5 in PIN and PCa in TMAs stratified by histological category. (A) The YY1 IOD and nuclear staining expression, and the DR5 IOD and DR5 protein expression in the cytoplasm and membrane, determined by immunohistochemistry, are shown as means represented as a bar graph, from the data of 270 informative tissue microarray spots containing NL (n=54), LG PIN (n=20), HG PIN (n=19), LG PCa (n=27), and HGPCa (n=27). (a) The mean YY1 IOD was significantly higher in the LG PCa (IOD=3675, P<0.001) and highest in the HG PCa (IOD=9042 P<0.001) as compared with the LG PIN (IOD=45) and the HG PIN (IOD=332). (b) The mean DR5 IOD was significantly lower in the LG PCa (IOD=3675 P<0.001) and significantly lower or absent in the HG PCa (IOD=9042, P<0.001) as compared with the LG PIN (IOD=45) and the HG PIN (IOD=332). (c) The mean YY1 expression in nuclear staining was significantly higher in the LG PCa (IOD=3675, P<0.001) and highest in the HGPCa (IOD=9042, P<0.001) as compared with the LG PIN (IOD=45) and the HG PIN (IOD=332). (d) The mean DR5 expression in the cytoplasm membrane was significantly lower in the LG PCa (IOD=3675, P<0.001) and significantly lower or absent in the HG PCa (IOD=9042, P<0.001) as compared with the LG PIN (IOD=45) and the HG PIN (IOD=332). (B) YY1 and DR5 boxplots on the PIN and the PCa of different degrees in the TMAs, stratified by histological category. The distribution of the study population into different groups is represented graphically. (a) YY1 IOD distribution in the LG PIN, HG PIN, LG PCa and HG PCa. Both the PIN box plots are short since the YY1 IOD had a homogeneous distribution and minimal dispersion of cases. The YY1 IOD was low in both groups. The YY1 IOD expression in the PCa groups had a box plot greater than the PIN groups. The YY1 IOD was higher in both groups. The LG PCa box plot was less large, with a negative asymmetrical distribution, indicating that the majority of the measurements were lower than the HG PCa, but higher than the PIN groups. In addition, there was a low dispersion due to the homogeneity of the measurements. The HG PCa box plot had a positive asymmetrical distribution, meaning that the majority of the measurements were higher than those of the other groups. All the differences were statistically significant with P<0.0001. (b) DR5 IOD distribution in LG PIN, HG PIN, LG PCa and HG PCa. Both PIN box plots were shorter than those of the YY1 IOD, most markedly in the HG PIN group, where measures were greater. The dispersion in both groups was minimal and although there was asymmetry, this indicated that the measurements were homogeneous. The DR5 IOD expression in the PCa groups was very low, with a low dispersion, and a symmetrical and homogeneous distribution, which was due to low or absent expression. All differences were statistically significant with P<0.0001. ^*^P=0.002 and ^**^P=0.01. YY1 Yin Yang 1; Dr5, death receptor 5; PIN, prostatic intraepithelial neoplasia; PCa, prostate carcinoma; TMA, tissue microarray constructions; IOD, integrated optical density; LG PCa, low grade prostate carcinoma; HG PCa, high grade prostate carcinoma; NL, normal prostate.

Using immunohistochemical analysis, the YY1 and DR5 expression in PCa tissue microarray samples was examined. The expression of YY1 (Fig. 1Aa and b; Fig. 1Ba and b) and DR5 (Fig. 1Ac and d; Fig. 1Bc and d) in human PCa tissues were examined in the PIN and malignant glandular epithelium (Figs. 1 and 2, respectively). YY1 was expressed predominantly in the nucleus and diffusely in the cytoplasm. DR5 was expressed on the membrane and in the cytoplasm. The expression of YY1 in the PIN samples was weak and predominantly localized to the cytoplasm. By contrast, the DR5 expression in the PIN samples was detected in the membrane and in the cytoplasm. In tumor samples, the YY1 expression was directly proportional to the malignant grade, whereby the highest expression was observed in HG PCa as compared with the expression in the LG PCa (Fig. 2Aa and b). By contrast, the DR5 expression was inversely proportional to the tumor grade (low in HG PCa and high in LG PCa) (Fig. 2Ac and d).

### Overexpression of YY1 and dowregulation of DR5 in PCa tissues

The expression of YY1 and DR5 was quantified by semiquantitative and quantitative assessments (Fig. 2B). There was a significant difference in the expression of YY1 between the LG PIN and HG PIN (Fig. 2Ba). In addition, there was a significant difference in the DR5 expression between the LG PIN and HG PIN samples (Fig. 2Bb) (P=0.001). YY1 was significantly elevated in PCa tissues as compared with the PIN tissues. YY1 expression was barely detectable in PIN tissues and was significantly increased (P<0.001, ANOVA) with an increase in tumor grade. This observation was similar in both the density quantification (Fig. 2Aa) and positive cell counts (nuclear) (Fig. 2Bc). An opposite effect was observed in the DR5 expression, whereby both the density of staining (Fig. 2Ab) and the positive cell counts (membrane) (Fig. 2Bd) were significantly decreased (P<0.001, ANOVA) as a function of the tumor grade. The highest levels of expression were observed in the PIN samples (Fig. 2Ac and d).

### Inverse correlation between DR5 and YY1 expressions in PCa tissues

A Pearson’s analysis was performed, based on the YY1 and DR5 expression in all of the tumor samples. The expression levels of YY1 and DR5 were found to be inversely correlated in both the LG PCa (P<0.036, r Pearson=−0.406) and HG PCa (P<0.0001, r Pearson=−0.611) samples (Table I).

### Bioinformatic analyses of YY1 and TNFRSF10B (DR5) gene expression in prostate adenocarcinoma

**Figure 3:**
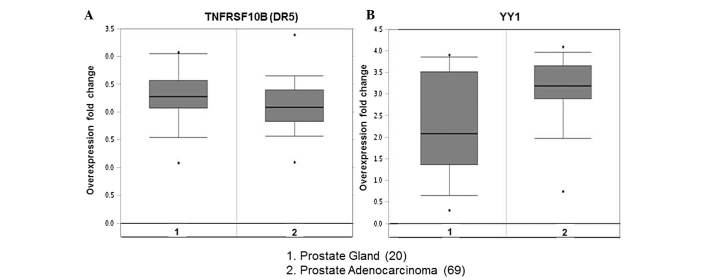
Bioinformatics analysis. The analysis of YY1 and DR5 mRNA expression levels in the prostate carcinoma was performed using a public dataset of microarrays retrieved from the Oncomine™ database and gene expression Omnibus. (A) Relative YY1 expression in normal and malignant cells. (B) Relative DR5 expression in normal and malignant cells. The Oncomine box plot of YY1 and DR5 expression levels are shown as boxed quartiles (median, 25th, and 75th percentile) and whiskers (minimum and maximum). ^*^P<0.05 by one-way analysis of variance. DR5, death receptor 5; YY1, Yin Yang 1.

Analysis of the TNFRSF10B (DR5) expression levels in different prostate tumors was performed using a public data set of microarrays retrieved from the Oncomine database and gene expression Omnibus, derived from the published analysis reported by Vanaja *et al* (28) and Wallace *et al* (29). The microarray data included 69 prostate adenocarcinomas and were compared with 20 samples from the prostate gland. In addition, YY1 and DR5 gene expression from the prostate adenocarcinoma samples were analyzed in the same data sets and compared with the prostate gland. The tumors showed low expression of DR5 as compared with the normal tissues (P=0.05 or 0.02, respectively). A higher expression of YY1 was observed in the same data sets as compared with the normal tissues (P<0.05) (Fig. 3). An inverse correlation of expression was observed between DR5 and YY1 in PCa. These findings were concordant with other database and gene expression analyses (30–33).

## Discussion

The present study has shown for the first time, to the best of our knowledge, the underexpression of DR5 in prostate cancer cell lines and patient-derived tumor tissues. The transcriptional regulation of DR5 was previously reported to be, at least in part, due to the repressor activity of YY1 (25). The present study therefore hypothesized that the transcriptional regulation of DR5 in human PCa would additionally be negatively regulated through the overexpression of YY1. The data of the present study confirmed this hypothesis and demonstrated an inverse correlation between the expression of DR5 and YY1. The experimental findings were corroborated by bioinformatic analyses. These data suggested that the expression levels of DR5 and YY1 in PCa may be novel prognostic factors in the progression of PCa.

The targeting of DR5 by TRAIL or anti-DR5 monoclonal antibodies has been the focus of various clinical trials in clinical cancers (34–36). The approach to inhibit the repression of DR5 through targeting YY1 will result in the upregulation of DR5 and its response to TRAIL-induced apoptosis. We have previously demonstrated that the inhibition of YY1 by YY1-specific siRNA in PCa cells resulted in sensitization of PCa cells to TRAIL apoptosis (37). It has been additionally shown that several anticancer agents overcome resistance to Apo2L/TRAIL through the upregulation of DR5 in malignant cells (25,38). In addition, the lower expression of DR5 has been noted in various types of cancer, including breast (39), lung (40), colorectal (41) and esophageal (42) cancers. Gene silencing of DR5 and DR4 was shown to abolish TRAIL-induced apoptosis (43).

Of note, the expression levels of DR5 have been described as a potentially useful prognostic marker in various cancers, including melanoma, where the decrease in DR5 expression was identified to be correlated with the progression as well as metastasis of the disease (20). Furthermore, DR5 expression was shown to be negatively correlated with the overall survival of breast cancer patients (22). DR5, however, was not found to be a significant prognostic marker in cervical or colon cancers (22,23). These observations suggested that the prognostic value of DR5 may be cancer type-specific. In PCa, the significance of DR5 expression had not been previously explored and the present findings suggested that it may be a significant novel prognostic marker in the progression of PCa.

The transcription factor YY1 is expressed in normal tissues and is upregulated in various types of cancer, including PCa, with positive and negative regulatory effects on gene expression. Elevated YY1 expression is correlated with the development of PIN and advanced prostate cancer (44,45). The results of the present study regarding the expression of YY1 and its correlation with the tumor grade are consistent with previous findings, which have linked higher YY1 expression to tumors, as compared with benign tissues.

In the present study, it was shown that YY1 was overexpressed in tumor samples as compared with PIN. The overexpression gradually increased with higher tumor grades (Fig. 2A and B). By contrast, DR5 was considerably higher in PIN as compared to tumors. This downregulation of DR5 in the tumors decreased further with increasing tumor grade (Figs. 1 and 2). In addition, Pearson’s analysis demonstrated an inverse correlation between the expression of YY1 and DR5 in PCa tissues (Fig. 3). This finding is consistent with the hypothesis that YY1 negatively regulates DR5 and this inverse correlation in PCa patient samples has an important implication in the development of this disease.

High DR5 expression has been linked to TRAIL-mediated apoptosis (15,25–27). DR5 expression was shown to be low in the tumors as compared with PIN, which suggested that the tumor cells may be resistant to TRAIL-mediated apoptosis. This is relevant since the TRAIL-DR5-mediated apoptotic pathway is involved in the immune-mediated apoptosis signaling response by cytotoxic T lymphocytes and natural killer cells (46). These results additionally suggested a mechanism whereby patient tumors with high YY1 expression and, therefore, low DR5 expression (repressed by YY1), would experience higher apoptosis resistance to both immune and chemotherapeutic drugs and, consequently, develop tumors of higher grades as a consequence of tumor unresponsiveness to therapy.

The present findings have only examined the expression of DR5 in PCa and several reports have demonstrated that DR5 is the preferential receptor-inducing signal for TRAIL apoptosis (25,27,39). The expression of DR4 and its role in TRAIL-induced apoptosis, however, cannot be ruled out. The expression of DR5 and DR4 is reduced in prostate tumors in comparison with that in benign tissues. Low expression of death receptors suggests resistance to apoptosis and, thus, increased tumorigenesis. However, YY1 has not been shown to regulate DR4 thus far to the best of our knowledge (47). Additional studies are therefore required to determine the role of DR4 in PCa and its clinical significance.

In conclusion, the present study demonstrated the clinical significance of DR5 downregulation in PCa and its inverse correlation with the expression of YY1. In addition, these data suggest a potential prognostic significance of both DR5 and YY1 in the progression of PCa.

## Figures and Tables

**Table I:** YY1 and DR5 expression and their correlation in PCa, LG PCa and HG PCa.

	IOD	PCa n=54	LG PCa n=27	HG PCa n=27
YY1	Median	5857.44	3676	8038
	range	2470-12446	2471-9622	3975-12446
	S.D.	3253	1429	3107
	Median	4570	3481	9128
	CI	4969	3111	6810
DR5	Median	132	193	60
	range	38-310	75-310	38-73
	S.D.	84	78.3	9
	Median	89	205	61
	CI	109	162	55
r Pearson YY1/DR5		−0.648	−0.406	−0.611
P-value		0.0001	0.036	0.001

P-values were determined by the Pearson χ^2^ test Yates continuity correction. The Pearson correlation was established using the percentage of nuclear YY1-positive and membrane protein DR5-positive specimens. Distribution of study population in PC, LG PCa and HG PCa. The correlation between YY1 and DR5 in PCa is −0. 648 with P=0.0001, in LG PCa is −0.406 (P=0.306) and in HGPCa is −0.611 (P=0.001). PCa, prostate carcinoma; LG PCa, low grade prostate carcinoma, Gleason scores 2 to 5; HG PCa, high grade prostate carcinoma, Gleason scores 6 to 10. DR5, death receptor 5; YY1, Yin Yang 1; S.D., standard deviation; IOD, integrated optical density; PIN, prostatic intraepithelial neoplasia; CI, confidence interval.
